# CD19 + CD23+ B cells, CD4 + CD25+ T cells, E-selectin and interleukin-12 levels in children with steroid sensitive nephrotic syndrome

**DOI:** 10.1186/1824-7288-39-42

**Published:** 2013-07-06

**Authors:** Bilal Yildiz, Nuran Cetin, Nurdan Kural, Omer Colak

**Affiliations:** 1 Department of Pediatric Nephrology, Faculty of Medicine, Eskisehir Osmangazi University, Eskisehir, TR 26480, Turkey; 2 Department of Biochemistry, Faculty of Medicine, Eskisehir Osmangazi University, Eskisehir, TR 26480, Turkey

**Keywords:** Steroid sensitive nephrotic syndrome, sCD19 + CD23+ B cells, sCD4 + CD25+ T cells, sE-selectin, Interleukin-12

## Abstract

**Background and methods:**

Soluble-lymphocyte subsets (sCD19 + CD23+ B cells and sCD4 + CD25+ T cells), soluble-adhesion molecules (sE-selectin) and interleukin-12 (sIL-12) were assayed to evaluate the pathogenesis of steroid sensitive nephrotic syndrome in 48 patients diagnosed with steroid sensitive nephrotic syndrome (SSNS) in active (AS) and remission stages (RS).

**Results:**

The ratios of soluble CD19 and sCD19 + CD23 increased in patients with AS with respect to the patients with RS and controls (p < 0.05). Increased sCD19 + CD23 ratios were preserved in the patients with RS when compared with the controls (p < 0.05). Moreover, the ratios of sCD4 + CD25 lymphocyte subsets were not significantly different among the groups. Similarly, serum sIL-12 levels were not considerably disparate between the AS and RS. Serum sE-selectin levels were higher in the patients with AS relative to the controls (p < 0.01) and RS (p < 0.05). No significant correlations were noted between sE-selectin and lymphocyte subset ratios, serum sIL-12 and immunoglobulin levels. There was a positive correlation between sE-selectin, triglyceride (r = 0.757, p < 0.0001) and cholesterol (r = 0.824, p < 0.0001) levels in patients with the AS.

**Conclusion:**

The present results indicate that the patients with SSNS appear to have abnormalities in sCD23 + CD19+ cells, defect in T regulatory cell activity, and injury in endothelial cells as indicated by the presence high sE-selectin. These abnormalities might play a role in the pathogenesis of nephrotic syndrome. sIL-12 seems to have no role in pathogenesis of nephrotic syndrome reflecting normal Th1 response.

## Background

Idiopathic nephrotic syndrome (INS) is the most prevalent kidney disease in children. Persistent immunogenic stimuli (such as viral infections, immunizations or allergens) might trigger nephrotic relapses in most of these patients. A primary immune disturbance is thought to be responsible for the pathogenesis of nephrotic syndrome in childhood. Various studies have attempted to identify potential abnormalities in lymphocyte subsets and they reported that during relapses, the subsets of CD4^+^ and CD8^+^ T cells expanded and levels of their cytokines increased (interleukin-2, IL-4 and interferon-) in the patients with nephrotic syndrome but reports regarding these measurements are conflicting [1-4]. Although steroid sensitive idiopathic nephrotic syndrome is a T lymphocyte mediated disorder, the pathogenetic role of B lymphocytes, effect of cytokines and vascular endothelial dysfunctions have not been well established in nephrotic syndrome. Therefore, in the present study we aimed to investigate the serum levels of soluble-lymphocyte subsets (sCD19 + CD23+ B cells and sCD4 + CD25+ T cells), soluble-adhesion molecule (sE-selectin) and interleukin-12 (sIL-12) in patients with steroid sensitive nephrotic syndrome (SSNS).

## Materials and methods

### Patients and control subjects

We included 48 patients diagnosed with SSNS (32 boys, 16 girls; age range 30–202 months) in the present study. The control group contained 19 healthy individuals (12 boys, 7 girls; age range 27–190 months). The patients were divided into two groups: 28 (58.3%) patients (20 boys, 8 girls) with active stage (AS) were grouped as Group 1 at the time of the diagnosis and 20 (41.7%) patients (12 boys, 8 girls) with remission stage (RS) were grouped as Group 2. Blood samples were collected before steroid treatment in Group 1. The patients who did not response the steroid treatment excluded from the study. The patients in Group 2 were selected among the steroid sensitive nephrotic patients at remission stage. The mean duration of treatment with steroids was 28 weeks in Group 2. The patients showing complications of nephrotic syndrome including infection, thromboembolism, osteoporosis or receiving blood transfusions, immunosupresive agents such as cyclosporin and cyclophosphamide, angiotensin-converting enzyme inhibitors, non-steroidal anti-inflammatory drugs and anti-histamines were excluded from the present study.

Active stage was defined as increased urinary protein excretion >40 mg/m^2^/h on timed sample or > 3+ by dipstick for 3 consecutive days, spot albumin to creatinine ratio >2 mg/mg and hypoalbuminaemia <2.5 g/dl). Remission stage was defined as urinary protein excretion <4 mg/m^2^/h; nil or trace by dipstick on spot sample for 3 consecutive days.

### Study protocol

The serum levels of E-selectine and IL-12 + p40 were measured in the patients with AS before steroid treatment and in RS and in the controls using commercially available kits (BioSource International, Inc. Camarillo, California 93012 USA). Assays were performed using solid phase sandwich ELISA. The blood samples for sE-selectine, and sIL-12 were kept at −70°C until the time of assay. Hemoglobin, erythrocyte count, platelet count, fibrinogen, total protein, cholesterol, triglycerides and albumin concentration were measured using standard laboratory methods.

Soluble peripheral lymphocyte subsets (CD3, CD4, CD8, CD19, sCD19 + CD23+ B cells and sCD4 + CD25+ T cells) were determined using double color flow cytometry (FACScan, Becton Dickinson, Sunnyvale, CA).

### Statistical analysis

Data were analyzed using the SPSS for Windows package. All ranges quoted represent the standard error or deviation. Mann–Whitney *U*-test, x^2^ test and Spearman's test were used for analysis. A p value <0.05 was considered to be statistically significant.

### Ethics

The current study was approved by the Research Ethics Committee of Eskişehir Osmangazi Medical Faculty, Eskişehir Osmangazi University. Informed consent was obtained from the parents or guardians of the patients and control subjects.

## Results

Overall, the IgG levels decreased and IgM levels increased in patients with the AS with regard to the controls and RS. Serum IgE levels also augmented in patients with the AS with respect to RS the patients and the controls. Increased levels of IgE were sustained in the patients with the RS when compared with the controls. The immunoglobulin levels are illustrated in Table 1.

**Table 1:** Serum Immunoglobulin (IgA, E, M and G) levels

	**Relapse (n = 28) (average + SD)**	**Remission (n = 20) (average + SD)**	**Control (n = 19)**	**p**
**IgA (mg/dl)**	93.9 ± 38.8	112.8 ± 70.1	134.5	p1 = NS
				p2 = NS
				p3 = NS
**E (mg/dl)**	392.5 ± 193.3	133.4 ± 32.8	32.4 ± 5.6	**p1 = 0.01**
				**p2 = 0.001**
				**p3 = 0.019**
**G (mg/dl)**	316.9 ± 237	741.1 ± 288.5	1093	**p1 = 0.002**
				**p2 = 0.0001**
				p3 = NS
**M (mg/dl)**	200.6 ± 121.9	121 ± 54	125	**p1 = 0.002**
				**p2 < 0.05**
				p3 = NS

p1: relapse vs. remission; p2: relapse vs. controls; p3: remission-controls.

Subset ratios of the soluble CD3 and CD8 lymphocyte were similar in all study groups. Subset ratio of the soluble CD4 lymphocyte decreased in patients with the AS with regard the patients with RS. Moreover, the ratios of soluble CD19 and sCD19 + CD23 increased as well in patients with AS in comparison to those with RS and controls (Table 2). Increased ratios of sCD19 + CD23 were present in patients with RS when compared with the controls. In addition, sCD4 + CD25 lymphocyte subset ratios were not notably different between the groups (Table 2). The ratios of lymphocyte subsets are summarized in Table 2.

**Table 2:** The soluble peripheral lymphocyte subsets ratios (%)

	**Relapse (n = 28) (average + SD)**	**Remission (n = 20) (average + SD)**	**Control (n = 19) (average + SD)**	**p**
**CD3**	65.8 ± 8.6	71.6 ± 6.3	69.4 ± 9.5	p1 = NS
				p2 = NS
				p3 = NS
**CD4**	30.8 ± 6.3	37.4 ± 6.9	35.8 ± 7.4	**p1 = 0.019**
				p2 = NS
				p3 = NS
**CD8**	27.2 ± 6.3	27.6 ± 7.1	27.8 ± 6.4	p1 = NS
				p2 = NS
				p3 = NS
**CD19**	20.3 ± 10.1	13.1 ± 4.2	12.8 ± 6.1	**p1 = 0.028**
				**p2 < 0.05**
				p3 = NS
**CD19 + CD23**	13 ± 9.7	7.7 ± 2.9	5.0 ± 2.3	**p1 < 0.05**
				**p2 = 0.04**
				**p3 = 0.046**
**CD4 + CD25**	4.8 ± 3.3	3.7 ± 1.4	3.6 ± 2.4	p1 = NS
				p2 = NS
				p3 = NS

p1: relapse vs. remission; p2: relapse vs. controls; p3: remission-controls.

**Figure 1:**
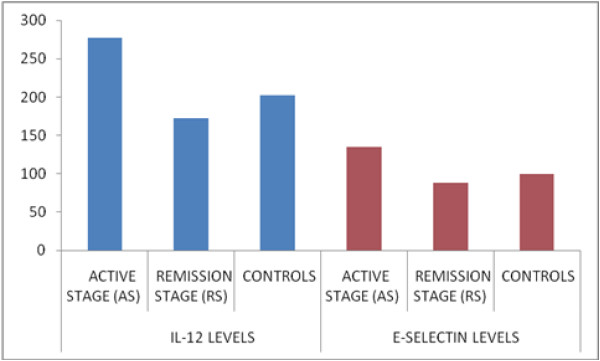
**Serum levels of sIL-12 and sE-selectin.** Serum levels of sIL-12 were similar in patients with active and remission stage and the controls. Serum E-selectin levels were higher in patients with active stage than remission satge and the controls.

In addition, serum sIL-12 levels were not considerably different between the two groups (Table 3, Figure 1). Serum sE-selectin levels were higher in patients with AS than controls and RS (Table 3, Figure 1).

**Table 3:** Serum soluble IL-12 and E-selectin levels

	**Relapse (n = 28) (average + SD)**	**Remission (n = 20) (average + SD)**	**Control (n = 19) (average + SD)**	**p**
**Soluble IL-12**	277 ± 185.8	172.3 ± 121.3	202.3 ± 45.3	p1 = NS
				p2 = NS
				p3 = NS
**soluble E-selectin**	134.8 ± 67,47	88.9 ± 38,8	100.2 ± 37.6	**p1 = 0.007**
				**p2 < 0.05**
				p3 = NS

p1: relapse vs. remission; p2: relapse vs. controls; p3: remission-controls.

**Figure 2:**
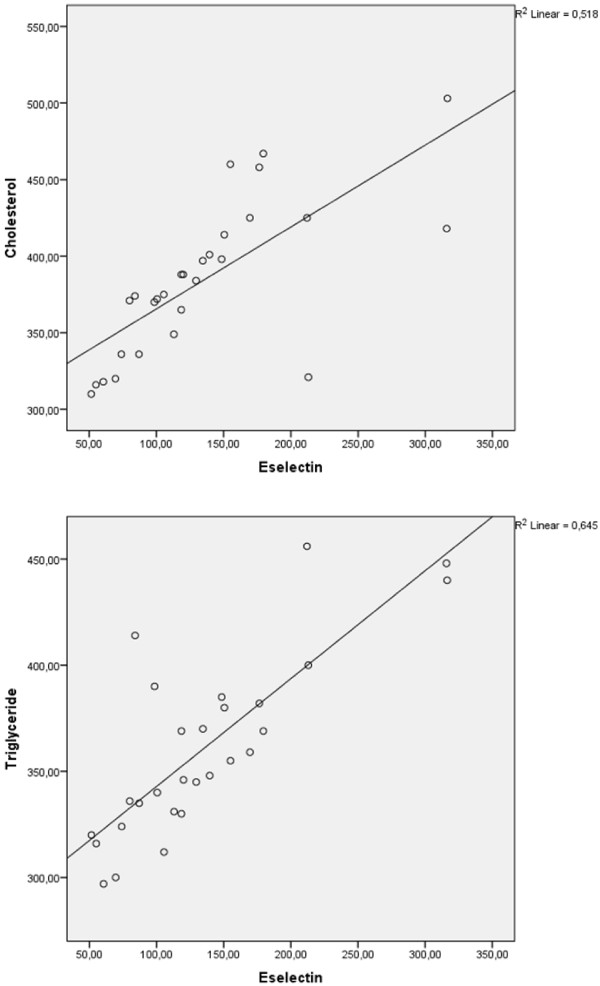
The positive correlations between E-selectin and triglyceride (r = 0.757, p < 0.0001) and cholesterol (r = 0.824, p < 0.0001) levels in nephrotic patients with active stage.

No significant correlations were noted between subset ratios of sE-selectin and lymphocyte, serum sIL-12 and immunoglobulin levels. There was a positive correlation between sE-selectin, triglyceride (r = 0.757, p < 0.0001) and cholesterol (r = 0.824, p < 0.0001) levels in Group 1 (Figure 2A and B).

## Discussion

The pathogenesis of INS is currently considered an immune mediated disease with particularly T lymphocyte involvement. Impairment in the immunoglobulin isotype switching, which is strongly T lymphocyte-dependent has been shown [5]. Similar to earlier studies, we measured markedly decreased IgG levels and considerably increased IgM levels during AS. However, in the present study these alterations did not statically persist in the patients with RS. According to our results, the low IgG levels might be related B cell disorders. We found low IgG levels and high CD19 and sCD19 + CD23+ B cell ratio which reflects increase in number or activity of B cells (Table 2). These findings suggest a defect in production of IgG despite of increased number of B cells.

The immune system deficiency in INS patients seems to be associated with excessive Th2 lymphocyte response. According to our findings, dominant T cell seems to be CD4+ T lymphocytes (Table 2). By contrast, Lama et al. report an imbalance of the CD4+/ CD8+ T lymphocytes distribution in favor of CD8+ T lymphocytes in relapse and remission in INS [6,7]. The existence of similar ratio of CD8+ T cells during in AS and RS and controls in the present study also suggested that CD8+ T lymphocytes were not dominant lymphocyte in patients with nephrotic syndrome. Our present observation is further supported by another study showing activation of the NFκB and c-maf transcription factors in CD4+ T lymphocytes during relapse [8]. Despite of increased CD4+ T cells, sCD4 + CD25+ T cells were not increased in our patients with AS. The absence of increased sCD4 + CD25+ T cells might result in increased Th2 response. In fact, there were increased IgE levels and similar sIL-12 levels in AS, RS and the controls which showed Th2 cell response in our patients with AS. Taken together, current study suggests that impairment of T regulatory cell (sCD4 + CD25+ T cell) is present in nephrotic patients.

The B lymphocyte anomalies have not been well studied so far in children with nephrotic syndrome. Our study suggests that lymphocyte impairments in nephrotic syndrome do not seem to be limited to the T cell. We found that there were more B lymphocyte expansion in AS and RS than that of the controls as indicated with increased sCD19 + CD23+ B lymphocytes and decreased IgG levels (Table 2). This finding prompted us to think that reduction of B lymphocytes could be preventing relapse ratios in patients with nephrotic syndrome. The therapeutic effects of rituximab, which suppresses B cell in the peripheral circulation as a chimeric anti-CD20 monoclonal antibody in nephrotic patients, supports our findings: (i) relapse rates following rituximab treatment may depend on the recovery of B-cells during the long-term course [9-11]. (ii) Moreover, rituximab might increase Treg frequency and number in patients with INS [12]. We did not find an increase in ratio of sCD4 + CD25+ T cells in patients with AS. Therefore, the increase of sCD4 + CD25+ Treg cells with rituximab supports our findings that Treg levels including sCD4 + CD25+ T cells is not enough in patients with nephrotic syndrome.

On the other hand, sCD19 + CD23+ B cells are related to allergic disorders [13]. sCD23 activation mediates IgE regulation, differentiation of B cells, activation of monocytes, and antigen presentation [14]. We found increased ratio of sCD19 + CD23+ B cells and serum IgE levels in AS and RS. These findings suggest that atopy and high IgE levels might not be related only with Th2 response in our patients. These findings also suggest that sCD19 + CD23+ B cells and associated increased serum IgE could be related to relapse of nephrotic syndrome due to continued high ratio of sCD19 + CD23+ B cells and IgE levels in patients with remission.

The sIL-12 is shown to be a master regulator of Th1 response and cell-mediated immunity and sIL-12 can also up-regulate the production of vascular permeability factor in INS [15]. Therefore, sIL-12 has been implicated in the pathogenesis of INS [15,16]. Despite of the presence of in vitro data obtained from culture supernatants, there is no enough information on serum levels of sIL-12 as far as we are aware of. We found that serum sIL-12 levels were statistically similar in AS, RS and the controls. Also, there were no correlation between sIL-12 and lymphocyte sub-population and serum IgE levels in our study. According to these findings, sIL-12 seems to have no role in pathogenesis of nephrotic syndrome in our patients. Macrophages and monocytes produce sIL-12 as an early response to antigenic stimuli; therefore, we think that in vitro sIL-12 production of lipopolisaccaride stimulated peripheral blood mononuclear cells of INS patients could not be specific for nephrotic syndrome [17]. Indeed, GATA-3 (Th2-specific transcription factor) related Th2 cytokines are shown to negatively influence the production of sIL-12 in patients with INS [18,19].

Adhesion molecules mediate the initial rolling of inflammatory cells along endothelial cells and platelets in response to pathological process. However, the role of adhesion molecules in the pathophysiology of nephrotic syndrome in children is not well known. Unlike other adhesion molecules, E-selectin is synthesized only by endothelial cells when activated by interleukin-1 or tumor necrosis factor- [20,21]. Thus, sE-selectin could be a candidate marker for detection of endothelial injury in nephrotic syndrome. We found that sE-selectin levels were increased in patients with AS and it returned to normal after treatment. To our best knowledge, these findings are the first report on sE-selectin in children with nephrotic syndrome. We think that endothelial injury is not related to Th2 response and B lymphocyte due to lack of any relationship between sE-selectin and lymphocyte subsets in our nephrotic patients with AS. These findings also cannot be explained with IL-12 levels which not increased in our patients with AS since IL-12 is demonstrated to increase the sE-selectin ligands on T lymphocytes [22]. The possible reason for increased E-selectin level might be the presence of hyperlipidemia which is reported to enhance the secretion of IL‒6 and TNF-alpha [23]. On the other hand, hypercholesterolemia has been reported to increase superoxide anion production in endothelial cells [24]. In fact, we found that cholesterol and triglyceride levels were positively correlated with E-selectin levels in our patients. Hyperlipidemia seems to be associated with endothelial damage.

Glucocorticoids are of proven benefit in the treatment of proteinuria in patients with SSNS. We found that sE-selectin levels decreased but high ratios of sCD19 + CD23+ B cells persisted with steroids therapy in patients with RS. Present findings suggest that steroid therapy can improve the endothelial cell functions but appears to fail to regulate expansion of B-cell in patients with RS. We think that expansion of the sCD19 + CD23+ B cells might contribute to the continuation of immune response in patients with RS. The implication of B-cells in SSNS remains to be investigated in detail in future studies.

## Conclusions

In summary, present study suggests that patients with SSNS have abnormalities in their sCD23 + CD19+ B cells and show endothelial injury with high E-selectin levels. Furthermore, sIL-12 seems to have no role in pathogenesis of SSNS which reflects normal Th1 response. Present findings also implied that the patients with SSNS have T regulatory cell defect as indicated by normal sCD4 + CD25+ T cell ratio despite the increased immune response including high levels of IgM, IgE, CD19 and expanded sCD23 + CD19+ B cells.

## Competing interests

The authors declare that they have no conflict of interests.

## Authors’ contributions

BY conceived of the study. BY and NK designed the study. OC made biochemical assay. BY, NC, OC and NK analyzed the data and critical revision of the manuscript for important intellectual content. BY drafted the manuscript and all authors read and approved of the manuscript.
